# Increased expression of miR‐641 contributes to erlotinib resistance in non‐small‐cell lung cancer cells by targeting NF1

**DOI:** 10.1002/cam4.1326

**Published:** 2018-03-01

**Authors:** Juan Chen, Jie‐da Cui, Xiao‐tong Guo, Xia Cao, Qing Li

**Affiliations:** ^1^ Department of Pulmonary and Critical Care Medicine The General Hospital of Ningxia Medical University Yinchuan 750004 China; ^2^ Ningxia Medical University Yinchuan 750004 China; ^3^ Cancer Center Daping Hospital and Research Institute of Surgery Third Military Medical University Chongqing 400042 China

**Keywords:** ERK signaling, erlotinib resistance, miR‐641, NF1, NSCLC

## Abstract

Epidermal growth receptor (EGFR)‐targeted tyrosine kinase inhibitors (TKIs) have emerged as first‐line drugs for advanced non‐small‐cell lung cancer (NSCLC) patients with EFGR mutations. However, most patients with NSCLC show acquired resistance to EGFR‐TKIs, and low expression of NF1 is a mechanism of EGFR‐TKI resistance in lung cancer. However, the mechanism by which NF1 is downregulated in EGFR‐TKI‐resistant NSCLC is unclear. Here, we found the increased expression of miR‐641 in NSCLC cells and human NSCLC samples with resistance to TKI compared to those with sensitive to TKI. In addition, our *in vitro* experiments show that overexpression of miR‐641 induces TKI resistance in NSCLC cells. Furthermore, we identified that miR‐641 activates ERK signaling by direct targeting of neurofibromatosis 1 (NF1) in NSCLC cells. Our data show that overexpression of NF1 or silencing of ERK can block miR‐641‐induced resistance of NSCLC cells to erlotinib treatment. Importantly, our animal experiments show that combination of miR‐641 inhibition and erlotinib treatment can significantly inhibit erlotinib‐resistant NSCLC growth, inhibit proliferation and induce apoptosis compared to single‐drug treatment. Our findings suggest that increased expression of miR‐641 significantly contributes to erlotinib resistance development in NSCLC cells through activating ERK signaling by targeting NF1 and that inhibition of miR‐641 may reverse acquired resistance of NSCLC cells to erlotinib treatment.

## Introduction

Lung cancer is the most frequently diagnosed cancer and a leading cause of cancer‐related mortality worldwide 1. Non‐small‐cell lung cancer (NSCLC) comprises about 85% of all lung cancers, and the overall 5‐year survival rate is only 15% 2. Epidermal growth factor receptor (EGFR) mutation is the most common type of gene mutations detected in NSCLC 3, and EGFR also is an important therapeutic target in NSCLC therapy. In fact, EGFR tyrosine kinase inhibitors (TKIs) have become the standard treatment for NSCLC with EGFR mutations 4. However, although EGFR‐TKIs are initially very effective, resistance eventually arises in almost all patients, resulting in a modest overall survival benefit 1, 5. The most common acquired resistance mechanism to EGFR‐TKIs is a T7920M secondary mutation of the EGFR gene in *cis*‐position, as it accounts for ~50% of all TKI‐resistant lung cancer cases 6. Also, several non‐T790M‐mediated acquired resistance mechanisms identified in clinical specimens were obtained after EGFR‐TKI treatment failure, including activation of insulin‐like growth factor 1 receptor, amplification of MET and ERBB2, upregulation of the AXL receptor, small‐cell lung cancer transformation, and EMT 1, 6. However, the acquired TKI resistance mechanism is still unknown for about one‐third of TKI‐resistant lung cancer patients.

Neurofibromin 1 (NF1) is a GTPase‐activating protein that is a key negative regulator of the Ras signaling pathway, which negatively regulates MAP‐ERK kinase. Recently, a report showed that lower expression of NF1 was significantly correlated with primary and acquired resistance of lung adenocarcinomas to EGFR‐TKIs in patients 1. However, the mechanism by which NF1 is downregulated in EGFR‐TKI‐resistant NSCLC is unclear.

MicroRNAs (miRNAs) is a class of small noncoding RNA molecules (19‐22 nucleotides) that function by negatively regulating gene expression by binding to the 3`‐untranslated region (3`‐UTR). Accumulating evidences show that dysregulated miRNAs play important roles in cancer initiation and progression. Interestingly, recent studies show that dysregulated miRNAs also involved in the acquired resistance to EGFR‐TKIs in NSCLC 7, 8. For example, studies show that downregulated expression of miR‐17‐5p 9 and miR‐223 10 closely associated with EGFR‐TKI resistance in lung cancer, and overexpression of these miRNAs can overcome EGFR‐TKI resistance.

Here, we report that miR‐641 level was increased in EGFR‐TKI‐resistant NSCLC, and it can induce erlotinib resistance in NSCLC cells through activation of ERK signaling by direct targeting of NF1. In addition, our data show that overexpression of miR‐641 can overcome EGFR‐TKI‐resistant NSCLC cells to TKI treatment.

## Methods

### Establishment of erlotinib‐resistant cell line

Erlotinib‐resistant cells were established as described by Rho et al.^11^ Briefly, PC‐9 cells were exposed to 10 nmol/L of erlotinib (Selleckchem, Houston, TX) for 48 h, then washed, and cultured in drug‐free medium. When cell density was reached 80% confluent, cells were re‐exposed to increasing concentrations of erlotinib. Same step was repeated until cells were able to grow in 1 *μ*mol/L erlotinib. The established resistant cell lines (PC‐9/ER) were maintained in medium containing 1 *μ*mol/L of erlotinib.

### Human samples

Human samples were obtained from 18 patients with EGFR‐mutant NSCLC who developed acquired resistance to erlotinib at General Hospital of Ningxia Medical University. All patients received oral erlotinib 150 mg/day, and the treatment was continued until the disease progressed. The response of the patients was evaluated using the response evaluation criteria in solid tumors 1.1 (RECIST 1.1) criteria, and the resistance to TKIs in NSCLC patients was diagnosed according to guidelines that provided by Jackman et al. 12 Samples were collected from patients before to start erlotinib treatment and after diagnosed that patients are acquired resistance to erlotinib. Tissue samples were collected by biopsy. After explaining the research study and the related procedures, written informed consent was obtained from the patients. Characteristics of the patients were summarized in Table 1. This research was approved by the Research Ethics Board of the General Hospital of Ningxia Medical University.

**Table 1:** Demographic and clinical characteristics of the study subject

Case No.	Age (years)	Gender	Stage	Histopathology	TKI regimen	Mutation type	Erlotinib treatment time
1	69	F	IV	Adenocarcinoma	Second line	Exon 19 Del	6 months
2	56	F	IV	Adenocarcinoma	First line	Exon 19 Del	12 months
3	57	M	IV	Adenocarcinoma	Second line	Exon 21 L858R	6 months
4	55	F	IV	Adenocarcinoma	First line	Exon 21 L858R	9 months
5	39	F	IV	Adenocarcinoma	Second line	Exon 19 Del	14 months
6	61	M	IV	Adenocarcinoma	First line	Exon 19 Del	17 months
7	55	F	IV	Adenocarcinoma	Second line	Exon 21 L858R	11 months
8	63	F	IV	Adenocarcinoma	First line	Exon 21 L858R	6 months
9	56	M	IV	Adenocarcinoma	Second line	Exon 21 L858R	20 months
10	60	F	III	Adenocarcinoma	Second line	Exon 21 L858R	15 months
11	40	F	IV	Adenocarcinoma	First line	Exon 21 L858R	14 months
12	54	M	IV	Adenocarcinoma	Second line	Exon 21 L858R	13 months
13	49	M	IV	Adenocarcinoma	Second line	Exon 19 Del	10 months
14	55	F	IV	Adenocarcinoma	Second line	Exon 21 L858R	16 months
15	66	M	IV	Adenocarcinoma	Second line	Exon 19 Del	6 months
16	41	F	IV	Adenocarcinoma	Second line	Exon 21 L858R	4 months
17	38	F	IV	Adenocarcinoma	Second line	Exon 21 L858R	8 months
18	58	M	IV	Adenocarcinoma	Second line	Exon 21 L858R	17 months

### Cell viability assay

Indicated cells were transfected with indicated oligonucleotides or plasmid using lipofectamine 2000 (Life Technologies, Carlsbad, CA). After 24 h of transfection, cells were plated in a 96‐well plate at a density of 5,000 cells/well. After 12 h of cell seeding, cells were treated with indicated drugs for 48 h, then cell viability was determined using a CCK‐8 kit (Dojindo Laboratories, Kumamoto, Japan), according to the manufacturer's protocol. Control nucleotides, miR‐641 mimic, and miR‐641 inhibitor were purchased from RiboBio (Guangzhou, China). siRNA of ERK and NF1 was obtained from Santa Cruz Biotechnology.

### Clonogenic assay

Cells were transfected with indicated nucleotides. Twenty‐hour hours after transfection, the cells were seeded onto 24‐well cell culture plates at a density of 200 cells/wells. After 12 h, the cells were subsequently incubated with or without 0.01 *μ*M erlotinib, and the medium was changed every 3 days. After 12 days, the cell colonies were fixed with cold methanol, stained with 0.1% crystal violet for 30 min, washed, air‐dried, and photographed.

### Reverse transcriptase real‐time quantitative polymerase chain reaction analysis

Total RNA was isolated from cells and tissues using TRIzol reagent (Life Technologies) according to the manufacturer's protocol. miRNAs from serum were isolated using the miRNeasy Serum/Plasma Kit (Qiagen, Germantown, MD) following the manufacturer's instructions. Mature miR‐641 and the RNU6 endogenous control were analyzed using the TaqMan microRNA Assay Kit. The expression of miR‐641 was quantified in relation to the expression of RNU6. For analysis of NF1 expression, RT and PCR were performed with a High‐capacity cDNA Reverse Transcription Kit and QuantiTect SYBR Green PCR kit (Life Technologies), respectively. The expression of CRYAB was quantified in relation to the expression of *β*‐actin. Primers for NF1 were 5′‐CGAATGGCACCGAGTCTT AC‐3′ and 5′‐GACCAGTTGGACGAGCCC ‐3′ and for *β*‐actin were 5′‐AGAGCTACGAGCTGCC TGAC‐3′ and 5′‐AGCACTGTGTTGGCGTACAG‐3′.

### Western blot

A total of 30 *μ*g protein were separated on sodium dodecyl sulfate–polyacrylamide gel electrophoresis and transferred to nitrocellulose membranes. The membranes were blocked for 1 h in Tris‐buffered saline with Tween 20 (TTBS) containing 5% skim milk. Membranes were then incubated with primary antibodies against ERK, p‐ERK (Thr202/Tyr204), NF1, and actin for overnight at 4°C. Afterward, membranes were incubated with secondary antibodies conjugated to horseradish peroxidase (HRP) for 3 h at room temperature. After washing, bands of interest were analyzed using a LAS‐3000 luminescent image analyzer (Fujifilm, Tokyo, Japan), and quantification of Western blot analysis was performed using the Multi Gauge version 2.02 program (Fujifilm, Tokyo, Japan). All antibodies were obtained from Abcam (Cambridge, MA).

### Detection of apoptotic cells

Cells were transfected with indicated nucleotides. After 24 h, cells were reseeded in six‐well plates. After 12 h of cell seeding, cells were treated with or without 0.02 *μ*mol/L erlotinib for 24 h, then subjected to apoptotic cell analysis. Apoptotic cells were determined using flow cytometric analysis with the Annexin V‐FITC kit (CalbioChem, Shanghai, China) according to the manufacturer's instructions. Apoptotic cells in tissue were detected using an in situ cell death detection kit (Roche, Mannheim, Germany) according to the manufacturer's instruction. Cells were determined by counting three randomly chosen fields per section, determining the percentage of green signaling‐positive cells (apoptotic cell) per 100 cells at ×400 original magnification.

### Immunohistochemistry (IHC)

Tissue sections were deparaffinized in xylene and rehydrated using alcohol gradients, then washed, and incubated in 3% hydrogen peroxide (AppliChem, Darmstadt, Germany) for 30 min to quench endogenous peroxidase activity. After washing in phosphate‐buffered saline (PBS), the tissue sections were incubated with 5% bovine serum albumin in PBS for 1 h at room temperature to block nonspecific binding. The Ki‐67 primary antibody was applied to tissue sections overnight at 4°C. The following day, tissue sections were washed and incubated with secondary HRP‐conjugated antibodies for 1 h at room temperature. After careful washing, tissue sections were counterstained with Mayer's hematoxylin (Dako, Carpinteria, CA) and washed with xylene. Coverslips were mounted using Permount (Fisher, Pittsburgh, PA), and the slides were reviewed using a light microscope (Carl Zeiss, Thornwood, NY). Cells were determined by counting three randomly chosen fields per section, determining the percentage of DAB‐positive cells (Ki‐67) per 100 cells at x400 original magnification.

### Luciferase report assay

3`‐UTRs of NF1, containing the predicted miR‐641 target sequence, were amplified from human genomic DNA and cloned into the miRNA Expression Reporter Vector at *MluI* and *HindIII* sites. For the luciferase reporter experiments, the indicated cells were seeded onto 24‐well cell culture plates and cotransfected with the Renilla luciferase plasmid, and indicated reporter plasmids contain firefly luciferase. After 48 h of transfection, the luciferase activity was measured using the dual‐luciferase assay system according to the manufacturer's instructions. The luciferase activity was normalized to the activity of renilla luciferase.

### Animal experiments

Animal experiment was conducted using 6‐week‐old female nude mice. PC‐9/ER cells were transfected with empty plasmid or miR‐641 antisense expression plasmid. After 24 h of transfection, 1.5 × 10^7^ cells in 100 *μ*L of phosphate‐buffered saline were subcutaneously injected into each mouse (12 mice per group). When the mean tumor size reached approximately 100 mm^3^, each group divided into two groups and treated with or without erlotinib (25 mg/kg body weight) by daily gavage. The tumor size was monitored once per week with calipers. The tumor volume also measured using MRI (7.0‐T MRI, Bruker Biospec 70/20USR, Germany) at the end of animal experiments. Mice were treated with indicated drugs every 3 days for 4 weeks. After 4 weeks of treatment, mice were sacrificed and tumor weight was measured.

### Statistical analyses

All data are presented as the means ± SD. Differences between groups were determined using unpaired Student's *t*‐test or one‐way ANOVA using the SAS statistical software package version 6.12 (SAS Institute, Cary, NC). *P*‐values <0.05 were considered statistically significant.

## Results

### Increased expression of miR‐641 contributes to erlotinib resistance development in NSCLC cells

**Figure 1:**
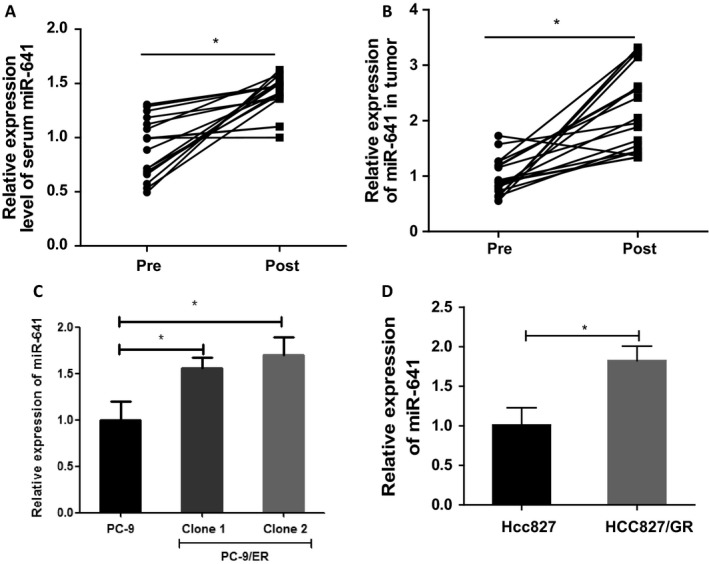
miR‐641 expression level was increased in EGFR‐TKI‐resistant NSCLC patients. (A) The level of miR‐641 was significantly increased in NSCLC patient serum that acquired resistance to erlotinib treatment (post) compared with matched pretreatment (pre). (B) The level of miR‐641 was significantly increased in NSCLC patient tumors that acquired resistance to erlotinib treatment (post) compared with matched pretreatment tumors tissue(pre). (C) The level of miR‐641 was significantly increased in erlotinib‐resistant cell PC‐9/ER compared to erlotinib‐sensitive cell PC‐9. (D) The level of miR‐641 was significantly increased in gefitinib‐resistant cell HCC827/GR compared to gefitinib‐sensitive cell HCC827. The levels of miR‐641 were measured by RT‐qPCR. *P <* *0.05.

**Figure 2:**
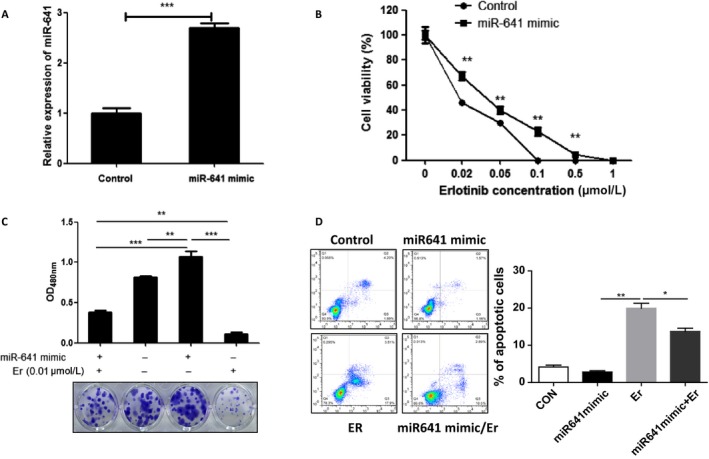
Overexpression of miR‐641 results erlotinib resistance in NSCLC cells. (A) Transfection of miR‐641 mimics significantly increased the miR‐641 level in PC‐9 cells. After 72 h of transfection, cells were subjected to qRT‐PCR analysis. (B) Cell viability assay shows that overexpression of miR‐641 can protect PC‐9 cells from erlotinib (Er)‐induced cell death. (C) Clonogenic assay shows that overexpression of miR‐641 abolished erlotinib‐induced colony formation inhibition of PC‐9 cells. (D) Flow cytometric analysis show that overexpression of miR‐641 protects PC‐9 cells from erlotinib‐induced apoptosis. *P <* *0.05; **P <* *0.01; ***P <* *0.001.

To investigate the correlation of miR‐641 expression level and EGFR‐TKI resistance in NSCLC patients, we measured the expression of miR‐641 in 18 human EGFR‐mutant NSCLC samples that acquired resistance to erlotinib treatment compared with matched pretreatment samples. As shown in Fig. 1A and B, serum and tumor analysis results show that miR‐641 expression was significantly increased in NSCLC patient samples with erlotinib resistance to erlotinib treatment compared to matched pretreatment samples. Consistent with these results, *in vitro* data also show that miR‐641 expression was significantly increased in erlotinib‐resistant NSCLC cell PC‐9/ER compared to their parental cell PC‐9 (Fig. S1A and C). Also, increased expression of miR‐641 was identified in gefitinib resistance NSCLC cell line HCC827/GR compared to their parental ell HCC827 (Fig. S1B and D), suggesting that increased expression of miR‐641 may be involved in EGFR‐TKIs resistance development of NSCLC cells. To investigate whether increased expression of miR‐641 affects sensitivity of NSCLC cells to erlotinib treatment, miR‐641 overexpressed PC‐9 cells were treated with erlotinib and then performed cell viability assay. As expected that overexpression of miR‐641 (Fig. 2A) significantly protected PC‐9 cells from erlotinib treatment‐induced cell death (Fig. 2B). Further, we confirmed this result using colony formation assay and observed similar results with cell viability assay (Fig. 2C). Consistent with these results, apoptosis analysis also show that overexpression of miR‐641 protects PC‐9 cells from erlotinib‐induced apoptosis (Fig. 2D). Taken together, these findings suggest that increased expression of miR‐641 significantly contributes to resistance development of NSCLC cells to erlotinib.

### miR‐641 causes erlotinib resistance via activating ERK signaling in NSCLC

**Figure 3:**
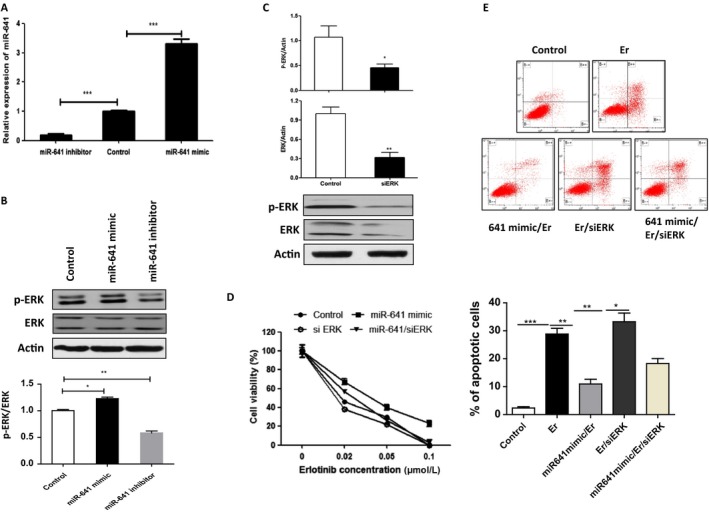
Overexpression of miR‐641 induces erlotinib resistance in NSCLC cells by activating ERK signaling. (A) Transfection of miR‐641 mimics or miR‐641 inhibitor significantly increased or reduced miR‐641 level in PC‐9 cells, respectively. After 72 h of transfection, cells were subjected to qRT‐PCR analysis. (B) miR‐641 positively regulates the phosphorylation of ERK in PC‐9 cells. PC‐9 cells were transfected with miR‐641 mimic or miR‐641 inhibitor (antisense of miR‐641). After 72 h of transfection, cells were subjected to Western blot analysis. (C) ERK expression was significantly inhibited by ERK siRNA treatment in PC‐9 cells. Cells were transfected with ERK siRNA. After 72 h of transfection, cells were subjected to Western blot analysis. (D) Silencing of ERK abolished miR‐641‐induced erlotinib resistance in PC‐9 cells. PC‐9 cells were transfected with miR‐641 mimics and/or siRNA of ERK. After 24 h of transfection, cells were treated with indicated concentration of erlotinib for 48 h, then subjected to cell viability assay. (E) Silencing of ERK‐enhanced apoptosis of miR‐641 overexpressed PC‐9 cells. PC‐9 cells were transfected with miR‐641 mimics and/or siRNA of ERK. After 24 h of transfection, cells were treated with indicated 0.02 uM erlotinib for 24 h, then subjected to cell viability assay. *P <* *0.05; **P <* *0.01; ***P <* *0.001.

Studies show that activated ERK‐MAPK signaling was closely correlated with development of EGFR‐TKI resistance in cancers 1, 13. Thus, we investigated whether miR‐641 affects ERK signaling in NSCLC cells. As expected, overexpression of miR‐641 significantly increased ERK phosphorylation in PC‐9 cells, while inhibition of miR‐641 reduced the phosphorylation of ERK (Fig. 3A and B), suggesting that ERK signaling may be involved in miR‐641‐induced erlotinib resistance of NSCLC cell. In fact, our cell viability assay shows that silencing of ERK (Fig. 3C) abolished that miR‐641‐induced erlotinib resistance in NSCLC cells (Fig. 3D). Consistent with this, apoptosis analysis data also show that silencing of ERK can abolish miR‐641‐induced protection effects of NSCLC cells to erlotinib treatment (Fig. 3E). Taken together, these data suggest that miR‐641 induces erlotinib resistance in NSCLC cells through activating ERK signaling.

### miR‐641 activates ERK signaling by direct targeting of NF1 in NSCLC cells

**Figure 4:**
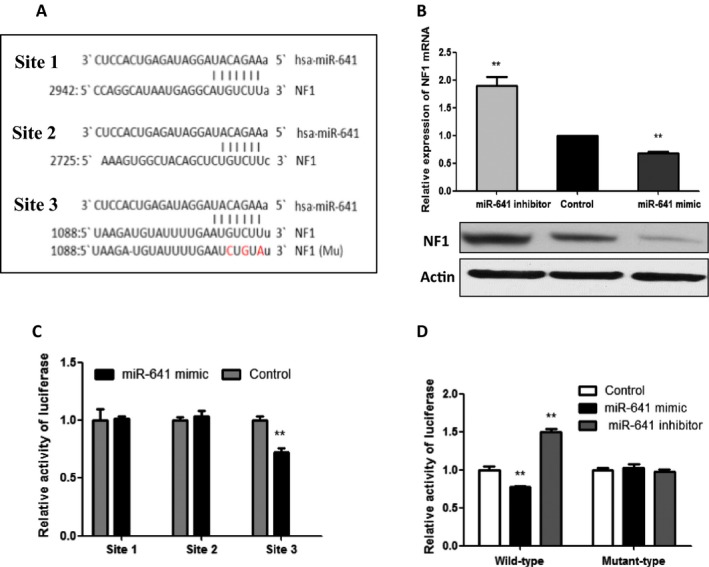
NF1 is a target gene of miR‐641. (A) Sequence alignment of miR‐641 with the 3`‐UTR of NF1 gene. (B) miR‐641 negatively regulates the NF1 expression at both mRNA and protein levels. PC‐9 cells were transfected with miR‐641 mimic or inhibitor. After 72 h of transfection, cells were subjected to RT‐qPCR and Western blot analysis. (C) A 3`‐UTR luciferase reporter assay for NF1. Each miR‐641 binding site of NF1 3`‐UTR was cloned into luciferase reporter constructs and transfected into PC‐9 cells with miR‐641 mimic or control oligonucleotides. After 48 h of transfection, the luciferase intensity was assessed. (D) NF1‐luciferase expression depends on the third binding site of NF1 3`‐UTR. The indicated plasmid was transfected with miR‐641 mimic or control oligonucleotides. After 48 h of transfection, cells were subjected to luciferase assay. *P <* *0.05; **P <* *0.01.

To investigate the underlying mechanism of miR‐641 on ERK signaling regulation, we screened miR‐641 target genes using miRNA target prediction algorithms and identified NF1 as a tentative target of miR‐641. Because the 3`‐UTR of NF1 gene contains three sites that can interact with miR‐641 (Fig. 4A) and previous study show that NF1 can inhibit ERK‐MAPK signaling in NSCLC 1. To investigate whether miR‐641 negatively regulates NF1 expression, we measured the NF1 expression in PC‐9 cells after overexpressing or inhibiting miR‐641. Our results show that miR‐641 negatively regulates NF1 expression in NSCLC cells at both mRNA and protein levels (Fig. 4B). Further, we investigated the direct binding of NF1 with NF1 3`‐UTR. To determine the direct interaction of miR‐641 with 3`‐UTR of NF1, we cloned each site of NF1 3`‐UTR that interacts with miR‐641 into a firefly luciferase reporter plasmid, than transfected to PC‐9 cells with miR‐641 mimic or control oligonucleotides. Our luciferase assay results show that transfection of miR‐641 mimic significantly decreased the luciferase activity in the group that transfected with third site of NF1 3`‐UTR (Fig. 4C), suggesting that miR‐641 directly interacts with the third site of NF1 3`‐URT. To confirm whether the regulation of NF1‐luciferase expression depends on the binding of their complementary 3`‐UTR sequences (third binding site) to the miR‐641 seed sequence, a three‐nucleotide mutation was inserted into the third targeting site of miR‐641 in 3`‐UTR, as indicated in Fig. 4A. Our data show that overexpression of miR‐641 significantly repressed the luciferase activity associated with the wild‐type 3`‐UTR. In contrast, the 3`‐UTR mutation completely abrogated the effect of miR‐641 overexpression on luciferase activity in PC‐9 cells (Fig. 4D).

**Figure 5:**
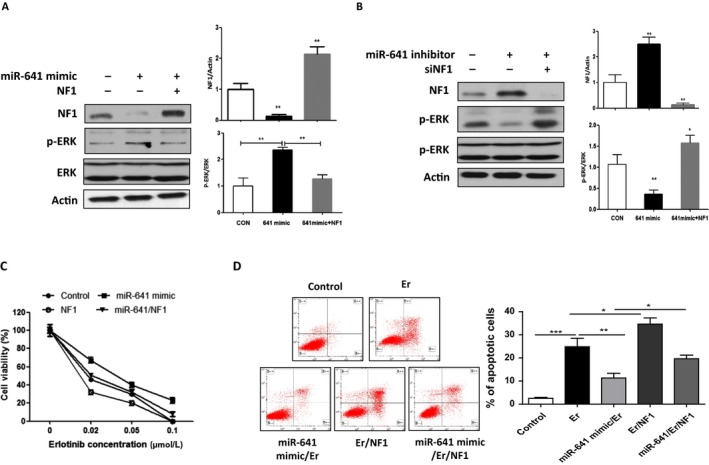
miR‐641 activates ERK signaling by inhibiting NF1 expression in NSCLC cells. (A) Overexpression of NF1 inhibits miR‐641‐induced ERK phosphorylation. PC‐9 cells were transfected with miR‐641 mimic or/and NF1 expression plasmid. After 72 h of transfection, cells were subjected to Western blot. (B) Silencing of NF1‐suppressed miR‐641 inhibitor induced inhibition of ERK phosphorylation. PC‐9 cells were transfected with miR‐641 inhibitor and/or NF siRNA. After 72 h of transfection, cells were subjected to Western blot analysis. (C) Overexpression of NF1 inhibits miR‐641‐induced erlotinib resistance in PC‐9 cells. Cells were transfected with indicated nucleotides and/or plasmid, then treated with indicated concentration of erlotinib for 72 h. (D) Overexpression of NF1 suppresses miR‐641 caused inhibition of erlotinib‐induced apoptosis in PC‐9 cells. Cells were transfected with indicated nucleotides and/or plasmid, then treated with 0.02 uM erlotinib for 24 h. *P < 0.05; **P < 0.01; ***P < 0.001.

Further, we investigated whether miR‐641 affects ERK signaling by regulating NF1 expression. As shown in Fig. 5A, overexpression of NF1 abolished miR‐641‐induced upregulation of phosphor‐ERK in PC‐9 cells. In contrast, silencing of NF1‐inhibited miR‐641 inhibition caused inhibition of ERK phosphorylation (Fig. 5B). In addition, our cell viability assay shows that overexpression of NF1 overcome overexpression of miR‐641‐induced erlotinib resistance in PC‐9 cells (Fig. 5C). Consistent with this, apoptosis analysis data also show that overexpression of NF1 can abolish miR‐641‐induced protection effects of NSCLC cells to erlotinib treatment (Fig. 5D). Taken together, these data suggest that miR‐641 induces erlotinib resistance of NSCLC cells through activation of ERK signaling by direct targeting of NF1 in NSCLC cells.

### Inhibition of miR‐641 can overcome resistance of NSCLC cells to erlotinib in vivo

**Figure 6:**
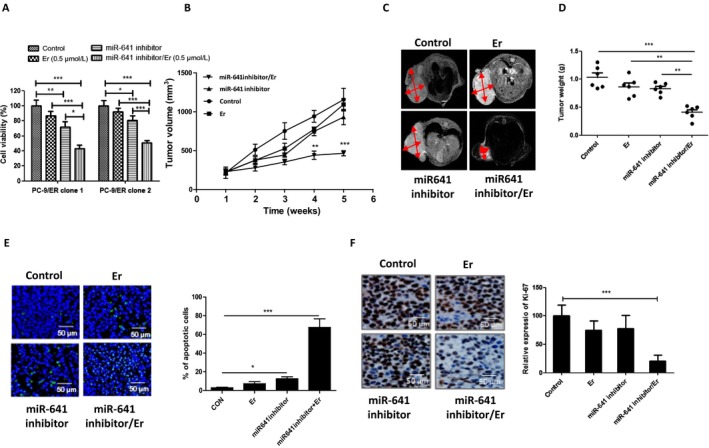
Inhibition of miR‐641‐enhanced sensitivity of erlotinib‐resistant NSCLC cells to erlotinib treatment *in vivo*. (A) Combination of miR‐641 inhibitor and erlotinib treatment synergistically inhibited PC‐9/ER cell viability. PC‐9/ER cells were transfected with negative control oligonucleotides or miR‐641 inhibitor for 24 h. Then, cells were reseeded in 96‐well plates. After 12 h of seeding, cells were treated with or without 0.5 μmol/L erlotinib for 48 h. (B) Combination of miR‐641 inhibition and erlotinib treatment synergistically suppressed tumor growth in PC‐9/ER xenograft models. (C) Tumor size was measured using MRI at the end of animal experiment. (D) Tumor weight. At the end of experiments, tumors were collected and measured the weight. (E) TUNEL assay shows that combination of miR‐641 inhibition and erlotinib treatment more significantly induces apoptosis in erlotinib‐resistant xenograft tumor. (F) Ki‐67 immunohistochemistry assay shows that combination of miR‐641 inhibition and erlotinib treatment more significantly inhibits cell proliferation in erlotinib‐resistant xenograft tumor. Er, erlotinib, *P < 0.05; **P < 0.01; ***P < 0.001.

Our observations that overexpression of miR‐641 induces erlotinib resistance in turn prompted us to investigate whether miR‐641 inhibition could overcome erlotinib resistance in NSCLC cells. To prove this hypothesis, we treated PC‐9/ER cells (from two different erlotinib‐resistant PC‐9 cell clones) with miR‐641 inhibitor (antisense of miR‐641) or/and erlotinib, then performed cell viability assay. Our results show that combination treatment of miR‐641 inhibitor and erlotinib more significantly inhibits cell viability compared to single‐drug treatment (Fig. 6A). Furthermore, to confirm these *in vitro* results *in vivo*, we, using miR‐641 inhibitor transfected erlotinib‐resistant cell line PC‐9/ER, generated xenograft model. When the mean volume of tumors is reached 100 mm^3^, mice were started to treat with or without erlotinib for 4 weeks. Our results show that treatment of erlotinib or miR‐641 inhibition (Fig. S2) cannot significantly suppress erlotinib‐resistant NSCLC tumor growth compared to control; however, combined treatment of erlotinib and miR‐641 inhibition significantly suppressed tumor growth compared to single treatment and control in PC‐9/ER xenograft model (Fig. 6B–D). Consistent with these results, TUNEL (Fig. 6E) and Ki‐67 IHC assay (Fig. 6F) results clearly show that combined treatment of erlotinib and miR‐641 inhibition more significantly promotes apoptosis and inhibit cell proliferation compared to single treatment. Taken together, these data suggest that inhibition of miR‐641 can overcome resistance of NSCLC to erlotinib.

## Discussion

EGFR mutation is one of the common types of gene mutation detected in lung cancer, and it also identified as an important therapeutic target 14, 15. Studies show that EFGR‐TKI treatment, such as erlotinib and gefitinib, can prolong the median progression‐free survival of 8‐16 months in the EGFR‐mutant NSCLC patients, but then, the EGFR‐TKI resistance may develop 16. Here, we identified that expression of miR‐641 was increased in NSCLC patients with EGFR‐TKI resistance compared to EGFR‐TKI‐sensitive NSCLC patients. In addition, we demonstrated that overexpression of miR‐641 can induce resistance of NSCLC cells to erlotinib. More importantly our *in vivo* experiment shows that inhibition of miR‐641 can overcome resistance of erlotinib‐resistant NSCLC to erlotinib. Taken together, these findings suggesting that increased expression of miR‐641 significantly contributes to EGFR‐TKI resistance development and inhibition of miR‐641 may be a novel strategy for treatment of erlotinib‐resistant NSCLC.

In this study, we also clarified the mechanism of miR‐641 on regulation of NSCLC cell sensitivity to erlotinib. In this study, we, using series *in vitro* experiments, identified NF1 as a target gene of miR‐641 in NSCLC cells. NF1 is a GTPase which converts active Ras‐GTP to its inactive form, thereby negatively regulates several signaling of Ras downstream, including Ras/MEK/ERK pathway 17, 18. In addition, previous study show that low expression of NF1 was associated with primary and acquired resistance of lung adenocarcinomas to EGFR‐TKIs in patients 1. Here, our data show that the restoration of miR‐641 expression in NSCLC cells leads to the suppression of NF1 expression and activates ERK signaling; conversely, inhibition of miR‐641 further upregulates NF1 expression and inactivates ERK signaling. In addition, luciferase reporter gene experiments show that miR‐641 directly targets the 3`‐UTR of NF1. Furthermore, our data indicate that restoration of NF1 blocks miR‐641‐induced ERK signaling activation; conversely, silencing of NF1 inhibited miR‐641 inhibition‐induced ERK signaling inactivation. Also, overexpression of NF1 or silencing ERK abolished miR‐641‐induced resistance of NSCLC cells to erlotinib. These data clearly suggest that decreased expression of NF1 is partially caused by increased expression of miR‐641 in erlotinib‐resistant NSCLC, and NF1 is a key downstream effector that mediates the effects of miR‐641 on NSCLC cell erlotinib sensitivity.

In summary, we first time determined the role of miR‐641 on the erlotinib resistance development in NSCLC cells. Our findings suggest that upregulated expression of miR‐641 was significantly associated with erlotinib resistance development in NSCLC cells. Our findings may also aid in the development of potential therapeutics for the treatment of erlotinib‐resistant NSCLC.

## Conflict of Interest

The authors have no conflict of interest.

## Supporting information


**Figure S1**. PC‐9/ER and HCC827/GR resistance to erlotinib and gefitinib treatment, respectively.

**Figure S2.** Transfection of miR‐641 inhibitor significantly suppressed miR‐641 expression level in PC‐9/ER xenograft tumor.
