# Correction to “Increased Expression of miR‐641 Contributes to Erlotinib Resistance in Non‐Small‐Cell Lung Cancer Cells by Targeting NF1”

**DOI:** 10.1002/cam4.70967

**Published:** 2025-05-19

Chen J, Cui J‐d, Guo X‐t, Cao X, Li Q. “Increased expression of miR‐641 contributes to erlotinib resistance in non‐small‐cell lung cancer cells by targeting NF 1.” Cancer Med. 2018; 7:1394–1403. https://doi.org/10.1002/cam4.1326.

In Figure 2D, an error occurred during the preparation of the figure, where incorrect representative flow cytometric plots were inadvertently selected. This has now been corrected and is shown below. The updated Figure 2D includes the correct representative plots, which accurately reflect the results of our experiment. Please refer to the revised figure for the accurate representation of the flow cytometric analysis. We would like to emphasize that this correction does not alter the conclusions of the study, as the error was limited to the selection of representative data and did not affect the interpretation or validity of the findings.

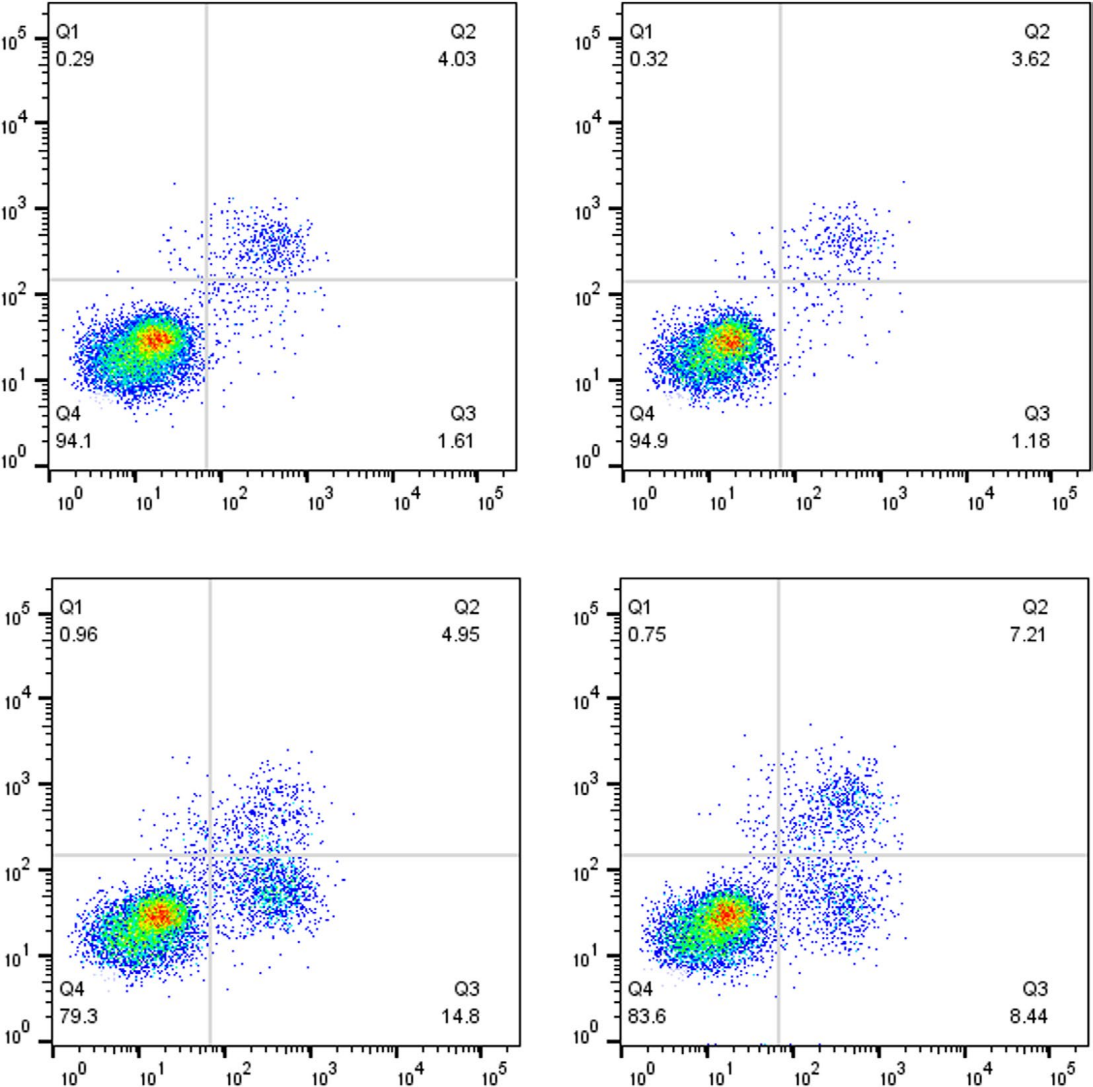

We apologize for this error.

