# Supplementary figures and images for: Increased expression of miR‐641 contributes to erlotinib resistance in non‐small‐cell lung cancer cells by targeting NF1
Source: Cancer Med. 2018 Mar 1;7(4):1394–403. doi: 10.1002/cam4.1326 (PMC5911582; doi:10.1002/cam4.1326)

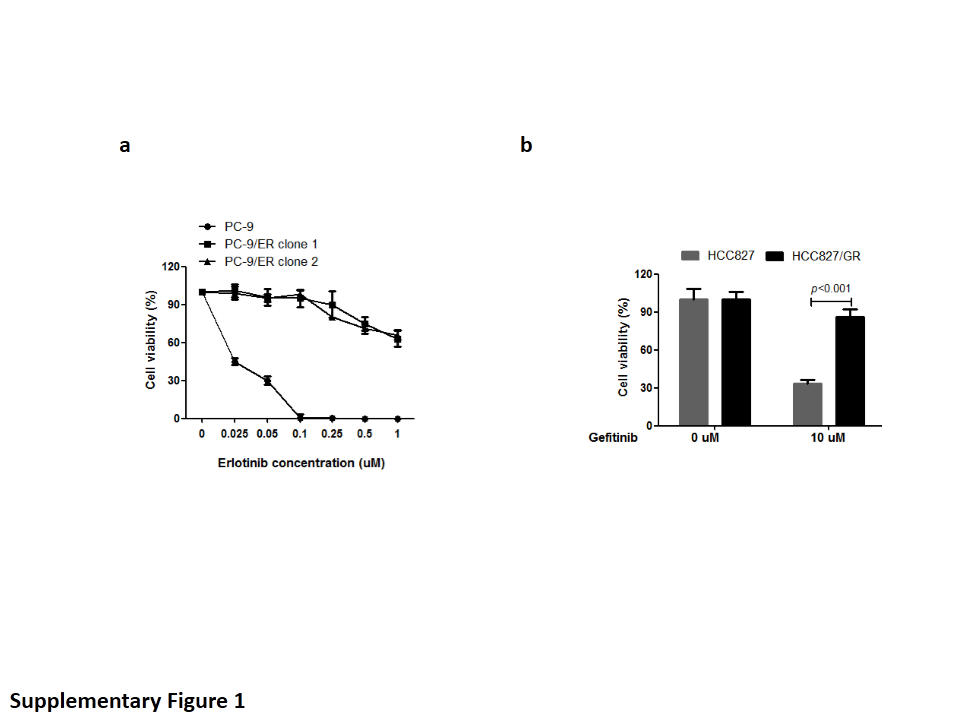

Supplement: Supplementary file 1 — Figure S1. PC‐9/ER and HCC827/GR resistance to erlotinib and gefitinib treatment, respectively. [file CAM4-7-1394-s001.TIF]

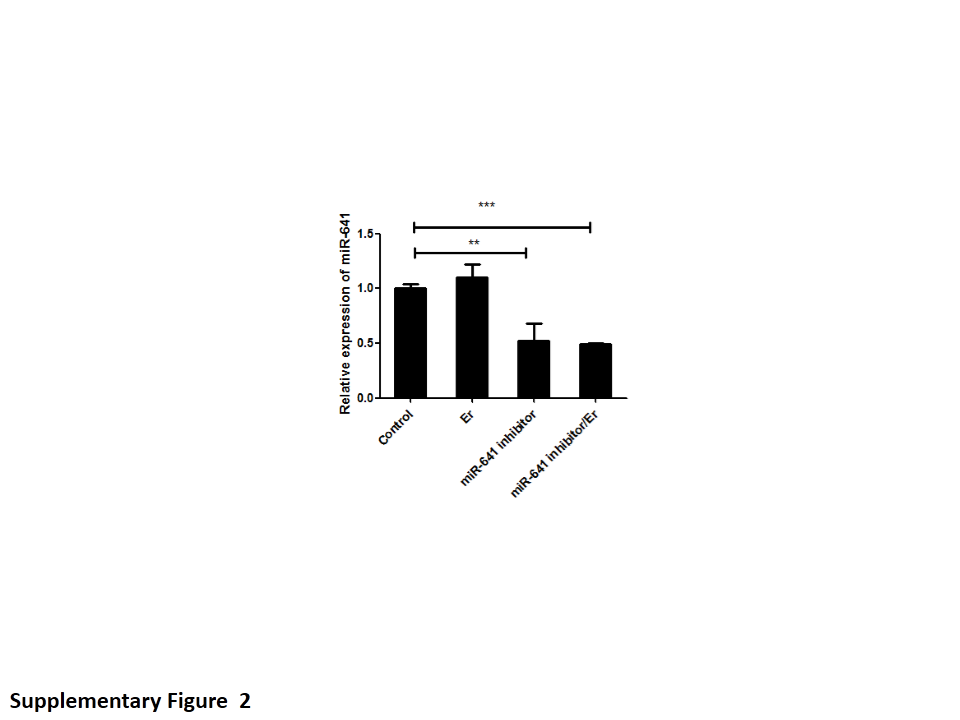

Supplement: Supplementary file 2 — Figure S2. Transfection of miR‐641 inhibitor significantly suppressed miR‐641 expression level in PC‐9/ER xenograft tumor. [file CAM4-7-1394-s002.TIF]
