# Supplementary material for: Increased expression of miR‐641 contributes to erlotinib resistance in non‐small‐cell lung cancer cells by targeting NF1
Source: Cancer Med. 2018 Mar 1;7(4):1394–403. doi: 10.1002/cam4.1326 (PMC5911582; doi:10.1002/cam4.1326)
Supplement: Supplementary file 3 [file CAM4-7-1394-s003.docx]

**Supplementary Figure legends**

**Supplementary Fig 1.** PC-9/ER and HCC827/GR resistance to erlotinib and gefitinib treatment, respectively. (**A**) PC-9/ER cells resistance to erlotinib treatment. (**B**) HCC827/GR resistance to gefitinib treatment. Indicated cells were treated with indicated drugs for 48 hours, then subjected to cell viability assay.

**Supplementary Fig 2.** Transfection of miR-641 inhibitor significantly suppressed miR-641 expression level in PC-9/ER xenograft tumor. PC-9/ER cells were transfected with empty vector or plasmid that expressing miR-641 antisense oligonucleotides, then subjected to xenograft generation. At the end of animal experiment, xenograft tumors were collected and the level of miR-641 was measured by qRT-PCR. **, *p* < 0.01; ***, *p* < 0.001.
